# Differing MODY subtypes in a single family: A case report on monogenic diabetes, a CARE-compliant article

**DOI:** 10.1097/MD.0000000000042315

**Published:** 2025-05-23

**Authors:** Sky C. Hart, Randy Pardue

**Affiliations:** a Lincoln Memorial University DeBusk College of Osteopathic Medicine, Knoxville, TN; b Family Medicine, Summit Medical Group, Knoxville, TN.

**Keywords:** case report, MODY, monogenic diabetes, pedigree

## Abstract

**Rationale:**

Mature Onset Diabetes of the Young (MODY) is a monogenic form of diabetes that presents distinct diagnostic challenges due to its clinical similarities with type 1 and type 2 diabetes. MODY results from specific genetic mutations affecting pancreatic beta cell function, and its inheritance pattern is autosomal dominant. This case report details a unique instance of differing MODY types within a single family, highlighting the diagnostic complexity and genetic variability of the disease.

**Patient Concerns:**

A 58-year-old male patient with unexplained hypercholesterolemia and hyperglycemia.

**Diagnoses:**

He was diagnosed with MODY type 1 by subsequent genetic testing revealing a mutation in the hepatic nuclear factor 4 alpha gene.

**Interventions:**

Treatment with glimepiride led to significant improvements in his metabolic profile.

**Outcomes:**

The patient’s diagnosis prompted genetic testing of his only child, who was found to have a different form of MODY, type 2, associated with a *glucokinase* gene mutation. This finding of differing MODY subtypes within one family is particularly noteworthy, given the typically consistent genetic transmission of MODY.

**Lessons:**

This case underscores the importance of accurate genetic diagnosis in the management of MODY and suggests that genetic testing should be considered more broadly in families with a history of diabetes, even when the clinical presentations vary. The identification of different MODY subtypes within a single family not only enhances our understanding of the disease’s genetic diversity but also has significant implications for personalized treatment strategies.

## 1. Introduction

Mature Onset Diabetes of the Young (MODY), also known as Monogenic Diabetes, is a subtype of Diabetes Mellitus (DM) in which beta cells of the pancreas malfunction. Patients with MODY lack insulin resistance and autoantibodies, distinguishing it from classic types of DM. MODY is estimated to account for 2% to 5% of DM cases, but its resemblance to type 1 and type 2 DM often leads to misdiagnosis.^[1]^

Several gene mutations cause MODY, each with a different presentation. Mutations in Hepatic Nuclear Factor 4 Alpha (*HNF4a*) on chromosome 20 cause MODY type 1. *HNF4a* is utilized in both liver and pancreas. In the liver, it regulates apolipoprotein synthesis, yet paradoxically increases lipoprotein (a).^[2]^ The reason behind this lipid increase remains unclear. In the pancreas, *HNF4a* mutations result in reduced insulin secretion in response to elevated glucose levels, though the mechanism behind beta cell dysfunction is still unknown.^[2,3]^ MODY type 1 accounts for 5% to 10% of MODY cases.^[3]^

Mutations in glucokinase (*GCK*) lead to MODY type 2. *GCK* phosphorylates glucose to glucose 6 phosphate. Mutations in *GCK* raise serum glucose levels and the insulin secretion threshold. MODY type 2 is the most prevalent, comprising 30% to 60% of all MODY cases.^[3]^

## 2. Patient information

This report discusses a 58-year-old Caucasian male with late-diagnosed MODY type 1, leading to subsequent genetic testing of his only child, who was found to have MODY type 2. Given the autosomal dominant inheritance pattern of MODY, this variation is intriguing.

The patient presented to a family medicine practice in Eastern Tennessee with a history of unexplained hypercholesterolemia and hyperglycemia. He was referred here after 2 years of no change in his lipid panel. The patient had no significant past medical or surgical history, engaged in vigorous daily exercise, and adhered to a ketogenic diet. He was not on medications and never smoked.

## 3. Clinical findings

Physical exam was unremarkable without signs of diabetes: Monofilament testing was intact bilaterally, upper and lower reflexes +2 and muscle strength 5/5 throughout. His body mass index (BMI) was 21.5, with no areas of localized obesity. There were no irregular skin lesions or darkening.

## 4. Diagnostic assessment

Laboratory tests revealed elevated glucose, hemoglobin A1c (HbA1c), cholesterol, and low-density lipoprotein levels. Triglycerides, high-density lipoprotein, and insulin levels were within the normal range (Table 1). A Boston Heart Cholesterol Balance showed elevated levels of demosterol (68), lathosterol (22), campesterol (144), and beta-sitosterol to be (192) with an apoB of 1.

**Table 1:** Lab values for MODY type 1 patient (father).

	November 2020	May 2021	August 2021	October 2021	March 2022	May 2022	September 2022	December 2022		February 2023		July 2023	January 2024	July 2024	Reference range
Glucose (mg/dL)	123	119	–	110	110	111	116	–	Referral	103	Glyburide started	116	–	100	70–100
Insulin (uIU/mL)	3.9	3.0	–	2.6	2.6	2.5	4.0	–		1.1		–	1.7	3.4	2–20
HA1c (%)	6.1	6.0		5.9	6.1	–	5.9	6.3		6.2		5.8	5.6	5.6	<5.7
Cholesterol (mg/dL)	454	342	386	317	365	–	–	390		474		371	–	328	<200
Triglycerides (mg/dL)	57	43	48	50	58	–	–	43		53		67	–	50	<150
HDL (mg/dL)	88	90	88	85	87	–	–	91		106		85	–	80	40–150
LDL (mg/dL)	355	243	288	222	266	–	–	290		366		269	–	233	<130

This table illustrates the various lab values taken for the patient across various visits. Glucose, insulin, Ha1c, cholesterol, triglycerides, HDL, and LDL decrease from 2020 to 2024.

HbA1c = hemoglobin A1c, HDL = high-density lipoprotein, LDL = low-density lipoprotein, MODY = mature onset diabetes of the young.

Negative insulin autoantibodies ruled out type 1 diabetes. His genomic DNA was sequenced using next generation sequencing through GBinsight on an Illumina HiSeq instrument. GBinsight based the sequencing off Human Genome Assembly 19, evaluating for single nucleotide variants and insertions and deletions under 10 nucleotides at targeted regions. Genetic testing identified mutations in several genes, including *Apolipoprotein E (APOe) E2, APOe E4, Cholesteryl ester transfer protein, Ghrelin, SH2B adaptor protein 1, Solute carrier organic anion transporter family member 1B1, Fat mass and obesity-associated protein,* and *HNF4a*, the latter being diagnostic for MODY type 1. Specifically, a missense mutation in *HNF4a* (nucleotide change c.416C > T, amino acid change p. Thr. 139 Ile) was identified, with a ClinVar ID of 129240. The other results are as follows:

• *APOe E2* missense mutation, heterozygous, ClinVar ID: 441266, classification: strong risk.
• *APOe E4* missense mutation, heterozygous, ClinVar ID: 17864, classification: strong risk factor.
• Cholesteryl ester transfer protein missense mutation, heterozygous, ClinVar ID: 319999, classification: risk factor.
• *Ghrelin* frameshift mutation, heterozygous, ClinVar ID: unknown, classification: VUS-likely strong risk factor.
• *SH2B adaptor protein 1* missense mutation, homozygous, ClinVar ID: unknown, classification: risk factor.
• *SLCO1B1* upstream variant, homozygous, ClinVar ID: 225995, classification: risk factor.
• *Fat mass and obesity-associated protein* intron variant, homozygous, ClinCar ID: 217824, classification: risk factor.

## 5. Therapeutic intervention

The patient began glimepiride 4 mg daily to increase insulin secretion and decrease serum glucose. After 6 months, his HbA1c dropped by 0.5%. He continued this treatment for 15 months, with consistent reductions in lipids, HbA1c, and glucose levels (Table 1), while his BMI remained unchanged. At the time of this study, the patient has continued glimepiride treatment with no adverse effects.

## 6. Follow up and outcomes

**Figure 1.**
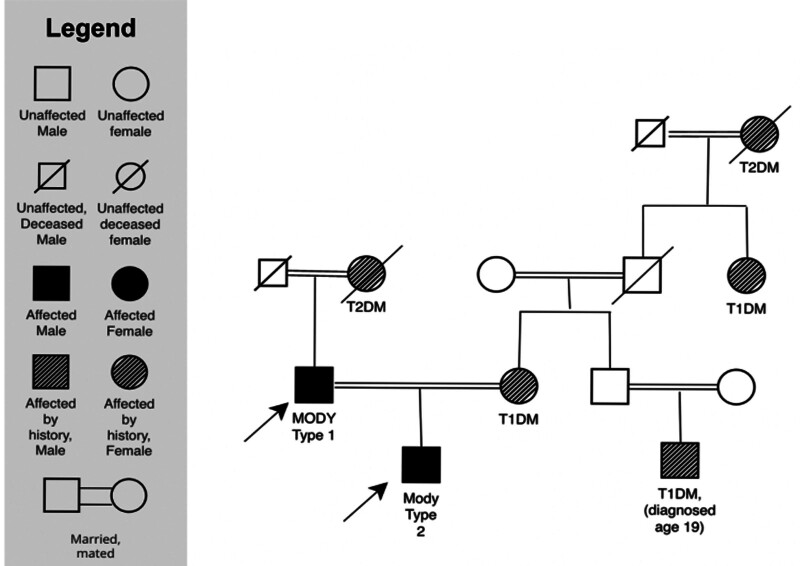
Family pedigree. The figure shows the father (Mody type 1) and son (Mody type 2) demonstrated by the arrows. The family history is based on historical report within the patients’ charts. The legend on the left shows the meaning behind the symbols.

After 1 year of treatment, they decided to test his only child due to the increased likelihood that he would receive the *HNF4a* mutation. He had no significant medical history. Family pedigree shown below (Fig. 1).

The child had no significant medical history and a BMI of 27.8, with no significant physical examination findings. Laboratory tests showed a glucose level of 113 mg/dL, insulin level of 9.5 µIU/mL, and HbA1c of 5.9%. His lipid panel showed elevated cholesterol (202 mg/dL), low-density lipoprotein (145 mg/dL), normal triglycerides (80 mg/dL), and low high-density lipoprotein (39 mg/dL). Genetic testing found a heterozygous for a missense mutation of *GCK* (c.951C > G, amino acid His317Gln), revealing a diagnosis of MODY type 2. This mutation leads to higher glucose levels and a reset insulin threshold, explaining the elevated fasting glucose despite normal insulin levels.^[3]^ The biological mother refused genetic testing at the time of this report, as she was diagnosed with type 1 diabetes mellitus (T1DM) via positive autoantibodies at age 27.

## 7. Discussion

The case underscores the importance of accurate diagnosis and the benefits of appropriate treatment. Development of diagnostic criteria for MODY genetic testing could enable earlier detection and intervention, potentially preventing the progression of symptoms.

MODY is often misdiagnosed as type 2 diabetes mellitus due to similar laboratory results. This study highlights the incidental discovery of MODY type 1 and the improvement that follows appropriate diagnosis and treatment. Despite its relatively low prevalence, MODY is believed to account for 1% to 5% of diabetes cases.^[4]^

By definition, MODY is present by age 25 in 95% of cases, however diagnosis can be missed until later in life. MODY is a monogenic disease with an autosomal dominant inheritance pattern, making the children of those diagnosed the highest risk group epidemiologically. Diagnosis is confirmed through genetic testing for mutations the 14 known causative genes.

Genetic testing should be considered for individuals who meet the following criteria:

- A child of a parent with a known MODY mutation.
- Non-T1DM, nonobese patients without signs of insulin resistance.
- Non-T1DM patients under 30 years of age.

For patients diagnosed with T1DM, it is recommended to monitor C-peptide and glucose levels over 3 to 5 years. If both remain preserved, genetic testing should be performed. In patients diagnosed with type 2 diabetes mellitus, there is no specific biological marker to differentiate MODY. Dr Ravi Kant has developed a flowchart in his paper, “Mature Onset Diabetes of the Young: Rapid Evidence Review,” which outlines the ideal pathway for MODY diagnosis.^[4]^

Treatment for MODY corresponds to its specific classification. Most cases can be managed with dietary changes and/or sulfonylureas. Since pancreatic beta cells still produce endogenous insulin, initiating sulfonylurea therapy is critical. Some patients may require additional exogenous insulin supplementation due to continued decline in pancreatic function.

Without proper treatment, patients with MODY type 1 are at risk for vascular complications, like other diabetic conditions. Additionally, due to MODY’s expression in the liver, there is a potential for lipid elevation and metabolic syndrome.^[3]^ Alternatively, MODY type 2 is seldom associated with such complications.^[3]^

Barriers to care for MODY patients often stem from misdiagnosis, particularly in those with higher BMI’s. Furthermore, genetic testing is not a routine diagnostic tool for DM, limiting access and affordability. However, with the increasing availability of genetic testing, there is hope that this barrier will diminish, leading to more accurate and timely diagnoses.

## Author contributions

**Conceptualization:** Sky C. Hart.

**Data curation:** Sky C. Hart, Randy Pardue.

**Formal analysis:** Sky C. Hart, Randy Pardue.

**Funding acquisition:** Sky C. Hart.

**Investigation:** Sky C. Hart.

**Methodology:** Sky C. Hart.

**Project administration:** Sky C. Hart.

**Resources:** Sky C. Hart.

**Software:** Sky C. Hart.

**Supervision:** Sky C. Hart, Randy Pardue.

**Validation:** Sky C. Hart.

**Visualization:** Sky C. Hart.

**Writing – original draft:** Sky C. Hart.

**Writing – review & editing:** Sky C. Hart.
